# The effect of hypertonic saline evoked muscle pain on neurophysiological changes and exercise performance in the contralateral limb

**DOI:** 10.1007/s00221-022-06342-6

**Published:** 2022-03-14

**Authors:** Ryan Norbury, Samuel A. Smith, Mark Burnley, Megan Judge, Alexis R. Mauger

**Affiliations:** Endurance Research Group, School of Sport and Exercise Sciences, University of Kent, Chipperfield Building Room 114, Canterbury Campus, Kent, CT2 7PE UK

**Keywords:** Muscle pain, Neuromuscular fatigue, Corticospinal excitability, Corticospinal inhibition, Sensory tolerance limit

## Abstract

Non-local muscle pain may impair endurance performance through neurophysiological mechanisms, but these are relatively unknown. This study examined the effects of muscle pain on neuromuscular and neurophysiological responses in the contralateral limb. On separate visits, nine participants completed an isometric time to task failure (TTF) using the right knee extensors after intramuscular injection of isotonic saline (CTRL) or hypertonic saline (HYP) into the left vastus lateralis. Measures of neuromuscular fatigue were taken before, during and after the TTF using transcranial magnetic stimulation (TMS) and peripheral nerve stimulation. Mean pain intensity was greater in the left leg in HYP (3.3 ± 1.9) compared to CTRL (0.4 ± 0.7; *P* < 0.001) which was combined with a reduced TTF by 9.8% in HYP (4.54 ± 0.56 min) compared to CTRL (5.07 ± 0.77 min; *P* = 0.005). Maximum voluntary force was not different between conditions (all *P* > 0.05). Voluntary activation was lower in HYP compared to CTRL (*P* = 0.022). No difference was identified between conditions for doublet amplitude (*P* > 0.05). Furthermore, no difference in MEP·*M*_max_^−1^ or the TMS silent period between conditions was observed (all *P* > 0.05). Non-local pain impairs endurance performance of the contralateral limb. This impairment in performance is likely due to the faster attainment of the sensory tolerance limit from a greater amount of sensory feedback originating from the non-exercising, but painful, left leg.

## Introduction

Pain is defined as an unpleasant sensory and emotional experience associated with actual or potential tissue damage (Raja et al. 2020). Muscle pain during exercise is caused by an accumulation of noxious biochemicals (Mense 2009) along with an increase in intramuscular pressure (O’Connor and Cook 1999). This sensation is referred to as exercise-induced pain, which increases as a function of exercise intensity (Cook et al. 1997) and time (Smith et al. 2020).

Exercise-induced fatigue can develop during exercise, which can be defined as a transient reduction in the maximal force generating capacity of the muscle that is reversible by rest (Gandevia 2001). Exercise-induced pain is often accompanied by exercise-induced fatigue (Pollak et al. 2014). Therefore, the two may be interrelated and consequently exercise-induced pain may at least in part be responsible for the development of fatigue (Mauger 2013) and be determinantal to endurance performance (Mauger et al. 2010; Astokorki and Mauger 2017; Stevens et al. 2018). Support for this notion comes from previous work which has found that muscle pain reduces the maximal force generating capacity of the painful muscle (Graven-Nielsen et al. 1997, 2002; Khan et al. 2011; Norbury et al. 2022) and impairs endurance performance (Ciubotariu et al. 2004; Smith et al. 2020). This effect appears to be driven by neurophysiological changes (Le Pera et al. 2001; Graven-Nielsen et al. 2002; Khan et al. 2011; Norbury et al. 2022) such as reductions in voluntary activation, corticospinal excitability and an increase in corticospinal inhibition. Because the fatigue related effects of muscle pain appear to be centrally mediated, it is possible that non-local muscle pain may also influence the development of neuromuscular fatigue in a non-local exercising limb, and subsequently reduce endurance performance (Hureau et al. 2018).

The effect of non-local pain on fatigue and endurance performance has recently been explored by Aboodarda et al. (2020). When ischaemia was induced on the left leg to gradually increase muscle pain, a 21% reduction in single limb cycling time to task failure (TTF) was seen in the right leg, which was coupled with lesser end-exercise reduction in maximum force and potentiated twitch force compared to no prior fatigue. Additionally, reductions in voluntary activation of non-local muscles have been found following a fatiguing protocol and subsequent maintenance of group III/IV afferent firing (and pain) through ischemia (Kennedy et al. 2013, 2014, 2015; Finn et al. 2020). Therefore, it is unclear how muscle pain may act at a non-local level to impact neuromuscular fatigue and endurance performance.

Intramuscular injections of hypertonic saline have previously been used to cause acute muscle pain (Graven-Nielsen et al. 2002; Khan et al. 2011; Smith et al. 2020, 2021). To explore the relationship between pain and fatigue, hypertonic saline may be advantageous to ischaemia as ischaemia traps blood in the occluded limb and lowers O_2_ availability. Furthermore, hypertonic saline induced pain can provide a different time course of pain intensity in comparison to ischemia, whereby saline produces a rapid increase, then slow decrease in pain intensity. Because of this, it is possible to determine the neurophysiological effects of non-local pain when exercise-induced pain and fatigue is low in the contralateral leg. Peripheral nerve stimulation and transcranial magnetic stimulation (TMS) are measurement techniques that can be used to investigate the neuromuscular function (NMF) of an individual in response to non-local muscle pain.

Therefore, the purpose of this study was to induce muscle pain in the left quadriceps and simultaneously assess endurance performance, neuromuscular fatigue, and corticospinal responses in the contralateral quadriceps. It was hypothesised that acute muscle pain would reduce endurance performance, and this would be accompanied by a decrement in the maximal force generating capacity as well as reductions in voluntary activation. Furthermore, we expected corticospinal excitability to be reduced and corticospinal inhibition to increase in response to muscle pain.

## Methods

### Participants

Twelve healthy individuals (3 female) with a mean ± SD age of 26 ± 5 years, height: 176 ± 9 cm, and body mass: 74.1 ± 13.0 kg participated in the study. A sample size calculation was performed to determine the number of participants required to detect a statistically significant effect in endurance time with pain. Based on an effect size for reduction in endurance time between control and contralateral pain which was *dz* = 1.09 from Aboodarda et al. (2020), an *n* = 9 was calculated to be needed to sufficiently detect an effect. All participants were free of lower limb injuries from the past 3 months, were not taking medication for the treatment of pain and had no pain related conditions. Participants were also screened for any contraindications to TMS (Rossi et al. 2011). Before testing commenced, all participants provided written informed consent and the study was approved by the University of Kent SSES research ethics advisory group (Prop 19_2019_20) and conducted in accordance with the declaration of Helsinki but without registering the study.

## Experimental protocol

**Fig. 1:**
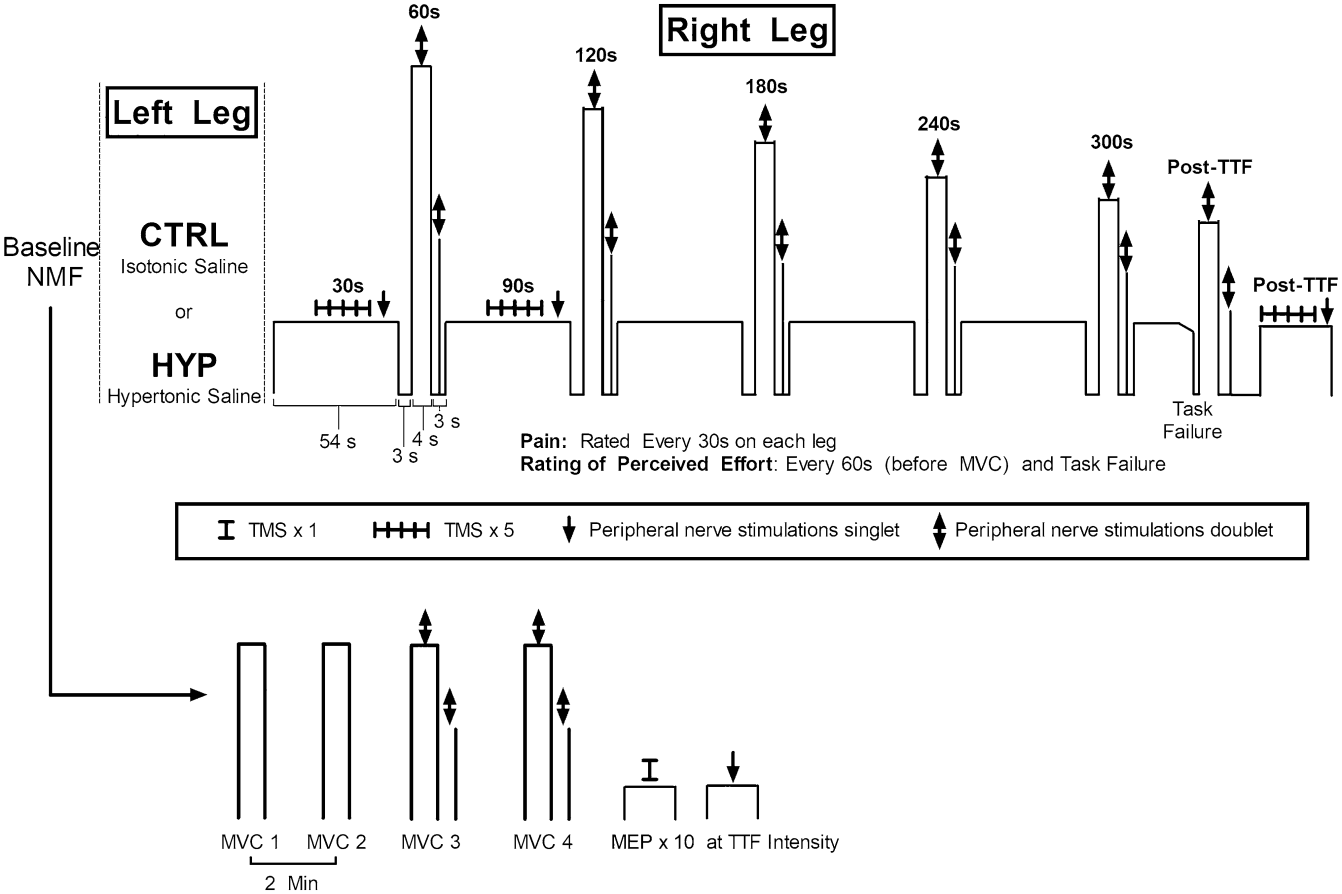
Schematic of the experimental protocol. RPE = rating of perceived effort, VL = vastus lateralis, NMF = neuromuscular function, TMS = transcranial magnetic stimulation

Participants visited the laboratory on four occasions separated by at least 72 h and at a similar time of day (± 2 h). Prior to visits, participants avoided strenuous lower body activity 48 h, caffeine 4 h, alcohol 24 h and analgesics 6 h. In the first visit, participants were familiarised with measures of neuromuscular function, questionnaires, perceptual measures, the isometric TTF exercise and the intramuscular injection of hypertonic saline (if they had not received one before, *n* = 6). Visit two comprised of a second familiarisation of the isometric exercise task where the intensity (% of maximum voluntary contraction) was adjusted from the first visit if the TTF was lower than four minutes or greater than 6 min. This was to ensure that task failure coincided with the typical pain duration from the intramuscular injection of hypertonic saline (Smith et al. 2020). Visits 3 and 4 comprised of the two experimental visits which were performed in a randomised order (see Fig. 1). Participants initially underwent baseline measures of NMF involving peripheral nerve stimulation and TMS during isometric contractions of the right knee extensors. Participants then waited ten minutes before receiving an intramuscular injection of 1 mL of isotonic saline (0.9%) or hypertonic saline (5.85%) in the left vastus lateralis (VL). The isotonic saline condition served as a non-painful, injection matched control (CTRL) while the hypertonic saline caused acute muscle pain (HYP). Immediately after the injection, participants began the submaximal isometric TTF protocol with the right leg whilst measures of peripheral nerve stimulation and TMS were performed. Measures of pain intensity and rating of perceived effort (RPE) were recorded until task failure, where post-exercise measures of NMF were performed along with the Situation Specific Pain Catastrophizing Scale.

### Equipment and procedures

#### Hypertonic saline injection

A bolus of 1 mL hypertonic saline (5.85% NaCl) was injected into the middle third of the muscle belly of the left VL to induce muscle pain. The site was cleaned with an alcohol swab and then the saline was manually infused using a 3 mL Luer-Lok syringe (BD, New Jersey, USA) connected to a 1.5 inch 25-gauge hypodermic needle (SurGuard2, Terumo, Japan) over a 20 s window (5 s pause after the insertion, a 10 s infusion period, followed by 5 s pause before needle withdrawal). An identical injection protocol was performed with the isotonic saline (CTRL condition).

#### Isometric endurance task

The endurance task was a single limb isometric contraction of the right knee extensors until task failure, which was defined as the inability to maintain the target force for 3 consecutive seconds despite verbal encouragement to return to the target. The intensity of the endurance task was individually prescribed to attain task failure in 4–6 min in CTRL (mean = 19% maximum voluntary force (MVF), range 14–25% MVF). At the end of each minute of the endurance task, participants were instructed to relax and to prepare to perform a 3 s maximum voluntary contraction (MVC) with a superimposed doublet. They were instructed to relax for a further 3 s after the MVC while a resting potentiated doublet was delivered. At 30 s and 90 s five TMS pulses were delivered (~ 3 s between stimulations). A schematic of the experimental protocol can be seen in Fig. 1.

#### Neuromuscular function testing

Baseline measures of NMF were completed after a warmup of ten contractions at 50% of the participants’ perceived maximum effort (3 s on, 3 s off). Four MVCs were then performed (3–5 s duration, 2 min rest between attempts). The latter two MVCs had a superimposed and resting potentiated doublet delivered during and after the MVC to determine voluntary activation. TMS pulses were delivered in a batch of 10 submaximal contractions at the same target force of the subsequent endurance task. One additional contraction was performed at the end of this batch with a single superimposed electrical stimulation. Within 10 s of cessation of the endurance task, another MVC with a superimposed doublet was performed along with 5 submaximal contractions with TMS and one contraction with a single electrical stimulation.

#### Mechanical recordings

Participants were strapped into a custom-built isometric chair with a hip and knee angle of 90° (0° being full extension). Straps secured the participant around the torso to prevent any extraneous movement and a non-compliant strap was secured 2 cm above the malleoli which was connected to a transducer to measure isometric force of the knee extensors. The transducer was connected to a signal amplifier (DA100c, Biopac Systems Inc, California, USA) and data acquisition module (MP150, Biopac Systems Inc, California, USA) and sampled at a frequency of 1.25 kHz in compatible software (Acqknowledge 5.0, Biopac Systems, California, USA). Force traces providing instantaneous feedback were displayed on a computer screen in view of the participant.

#### Electromyography (EMG)

Bipolar surface EMG was used to record muscle activity of the VL with 37.5 × 37.5 mm Ag/AgCl electrodes (Whitesensor 4831Q, Ambu Ltd, Denmark) at an inter electrode distance of 37.5 mm. The electrodes were placed on the muscle belly proximal to the knee and parallel with the fibres of the muscle. The site was shaved, abraded and cleaned to reduce impedance and the electrode locations were marked for replication in subsequent visits. All EMG data was recorded continuously at a frequency of 2.5 kHz and amplified (gain 1000 for VL, 2000 for BF) with a signal amplifier (EMG2-R, Biopac Systems, California, USA and EMG100c, Biopac Systems, California, USA) before being band pass filtered (10–500 Hz) and recorded onto compatible software (Acqknowledge v5.0, Biopac Systems, California, USA).

#### Peripheral nerve stimulation

An electrical stimulator (DS7r, Digitimer, Hertfordshire, UK) (maximum voltage = 400 v, pulse duration = 2 µs) capable of delivering a single square wave pulse was used for PNS. The anode was an adhesive electrode (100 mm × 50 mm; Phoenix Healthcare Products Ltd, Nottingham, UK) which was secured to the gluteal fold. Initially the cathode was a motor point stimulation pen (Motor Point Pen; Compex; DJO Global, Guildford, UK) which was placed within the femoral triangle to innervate the quadriceps femoris muscles. The motor point pen was used to identify the precise location within the femoral triangle which evoked the largest twitch force and compound muscle action potential (M-wave) peak to peak amplitude. A 32 × 32 mm electrode was placed over this site (Nessler Medizintechnik, Innsbruck, Austria) for all subsequent stimulations. To determine the intensity required to achieve supramaximal stimulation, 20 mA stepwise increments in stimulation intensity were delivered from 100 mA until a plateau in twitch force and M-Wave amplitude was observed. An additional 30% was added to ensure supramaximal stimulation (*M*_max_) (mean stimulator intensity = 214 ± 54 mA). Doublets were delivered as 100 Hz paired stimuli (1 ms pulse duration) for the assessment of central and peripheral fatigue whereas single stimuli (2 ms pulse duration) were delivered for the normalisation of MEPs.

#### Transcranial magnetic stimulation

Single pulse TMS was delivered with a magnetic stimulator (Magstim 200^2^, The Magstim Company Ltd, Carmarthenshire, UK) via a double cone coil (110 mm diameter) delivering a posterior-anterior current which was placed over the left motor cortex to assess cortico-spinal excitability of the right quadriceps muscle. Initially the participant’s vertex was marked as the midpoint between the nasal-inion and the tragus. The coil was placed 2 cm laterally to this position. Stimulations were superimposed over a submaximal contraction (same as target force for subsequent exercise; ~ 20% MVF) of the knee extensors at 50% of maximal stimulator output until the hotspot was found by moving the coil in 1 cm increments medio-laterally from the initial position, then anterior or posterior. The hotspot was defined as the location which provided the greatest peak to peak EMG amplitude of the motor evoked potential (MEP) in the VL. This location was marked on a skin-tight hat the participant was wearing. The participant also wore a cervical collar to prevent excessive movement of the head. Subsequent TMS pulses were separated by approximately 3 s which were superimposed over the submaximal knee extensor contraction. Stepwise increments in the stimulator intensity of 5% were used until a plateau in the average of the five MEPs was reached (< 5% increase) (mean stimulation intensity = 64 ± 8% maximum stimulator output). Each batch of TMS pulses (10 at baseline, 5 during exercise) were accompanied by the delivery of a single peripheral nerve stimulation to acquire MEP·*M*_max_^−1^ ratio.

#### Perceptual measures

To assess pain intensity, the pain perception scale was used (Cook et al. 1997) and participants rated their muscle pain for each leg every 30 s. The scale ranged from 0 which corresponded to ‘no pain at all’ to 10 which corresponded to ‘Extremely intense pain (almost unbearable)’. Participants were instructed to anchor the upper pain ratings to the worst exercise-induced pain they had previously experienced. Rating of perceived effort (RPE) was recorded on the 6–20 point scale (Borg 1982) to avoid participants conflating pain and effort ratings. Instructions were also given to participants to exclusively rate their effort based on the ‘effort to drive the limb’ with a rating of 20 anchored to the level of drive given during the MVC performed prior (Pageaux 2016).

#### Questionnaires

Post exercise, the Situation Specific Pain Catastrophizing Scale (Edwards et al. 2006) was administered.

### Data analysis

Baseline NMF was calculated as the mean of the raw value. MVF and doublet amplitude were calculated as the peak instantaneous force. Voluntary activation was calculated as (Strojnik and Komi 1998):

$$100 - {\text{SI Doublet}} \times \frac{{\left( {\text{force before SI doublet/peakforce}} \right)}}{{\text{resting potentiated doublet}}} \times 100$$

The mean of the MEP peak to peak amplitudes was normalised to the proximal M-Wave peak to peak amplitude to calculate the MEP·*M*_max_^−1^ ratio. The TMS silent period was calculated as the point from the stimulus artefact to the resumption of the EMG signal by the same investigator. The root mean square (RMS) of the electromyogram was calculated offline using a 100 ms time constant. MVC RMS amplitude was calculated as the 250 ms either side of peak force, whereas for the submaximal contraction amplitude the mean amplitude of 20 s at the start of each minute and 20 s before task failure was used. Both EMG variables (i.e., MVC and endurance task amplitude) were normalised to the baseline value and expressed as a percentage of max.

### Statistical analysis

All data are presented as mean ± SD or as mean and interquartile range when not normally distributed. Data was analysed in JAMOVI 1.6.7. Data was initially checked for assumptions with the Shapiro–Wilk test and the Mauchly test. If these assumptions were violated, data was analysed with a non-parametric test (Wilcoxon’s signed-rank) or Greenhouse-Geiser corrected, respectively. A 2 × 5 repeated measures analysis of variance (ANOVA) (condition × time) was used to analyse neuromuscular variables at baseline, minutes one, two, three, and task failure. A 2 × 4 repeated measures ANOVA (condition × time) was used to analyse TMS data. A 2 × 8 repeated measures ANOVA (condition × time) was used to analyse pain data. If an interaction effect was observed, followed-up post-hoc tests were performed to determine differences between conditions at different time points and were Bonferroni-Holm corrected (Holm 1979). Paired samples t-tests were used to test for differences in TTF. 95% confidence intervals, Cohen’s d effect sizes (ES) (Cohen 1992) where 0.2, 0.5 and 0.8 represent small, medium and large effect sizes, respectively and partial eta squared (${h}_{\mathrm{p}}^{2}$) were reported where appropriate and 0.01, 0.06 and 0.14 reflect small, medium and large effect sizes, respectively.

## Results

### Time to task failure

**Fig. 2:**
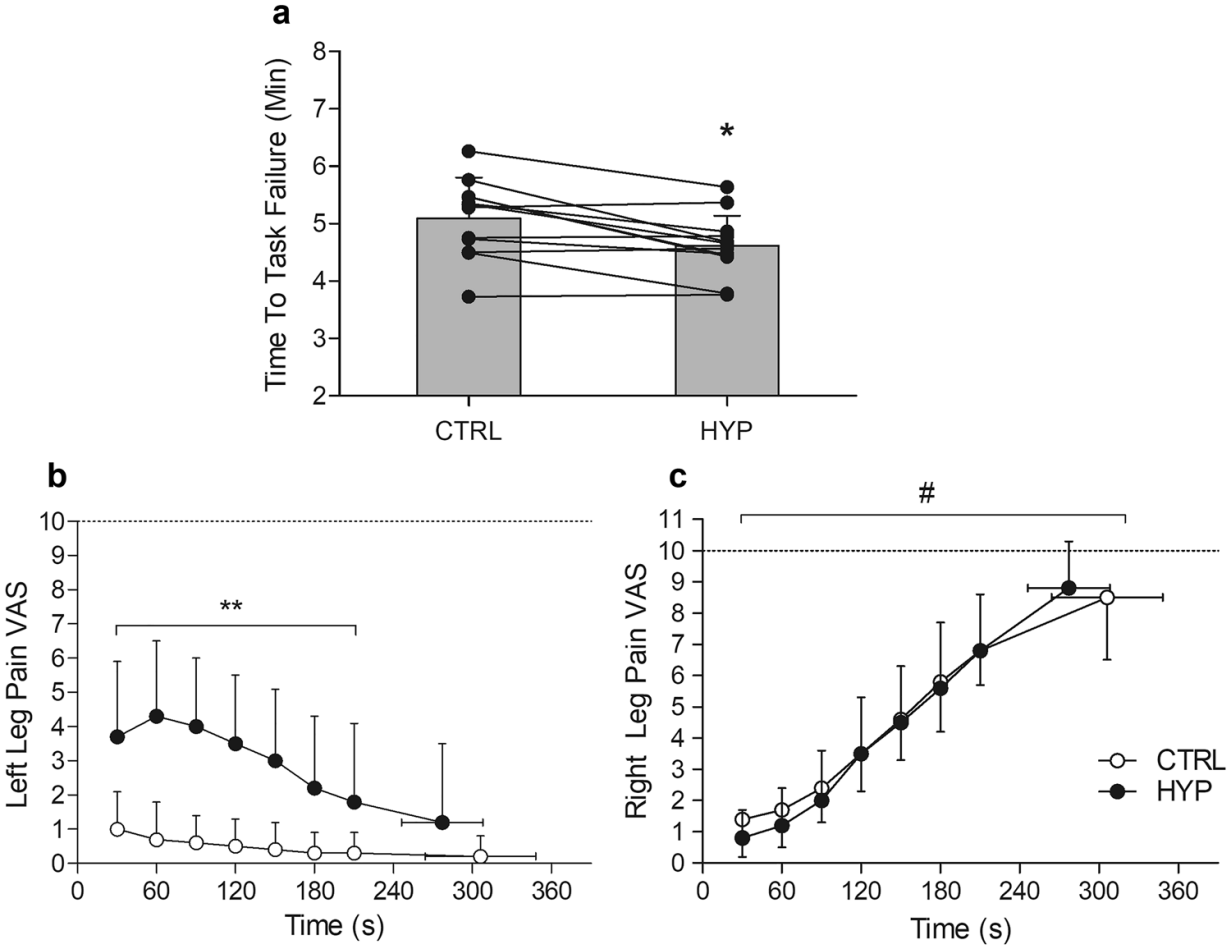
**a** TTF of the endurance task. * Denotes significantly different from CTRL (*P* = 0.005). Data presented as mean and individual data. **b** Left leg pain during the endurance task. ** denotes significantly different from CTRL (Interaction effect) (*P* < 0.001). **c** Right leg pain during the endurance task. # Denotes significant main effect of time (*P* < 0.05)

TTF was 9.3% shorter in HYP (4.62 ± 0.54 min) compared to CTRL (5.09 ± 0.68 min) (mean difference = 0.47 min, 95% CI [0.18, 0.76 min], *t*_11_ = 3.60, *P* = 0.004, *dz* = 1.04) (Fig. 2).

### Pain intensity

#### Left leg (saline-injected leg)

There was a condition × time interaction for left leg pain (*F*_2.03, 22.32_ = 8.53, *P* < 0.001, ${h}_{\mathrm{p}}^{2}$ = 0.437). Left leg pain did not change over time in CTRL (all *P* > 0.05), whereas in HYP, pain intensity was increased and greater than CTRL at all timepoints (all *P* < 0.01) except at 210 s (*P* = 0.088) and at task failure (*P* = 0.592) (see Fig. 2b). Peak pain was also greater in HYP (4.6 ± 2.2) than CTRL (1 ± 1.1) (mean difference = 3.6, 95% CI [2.2, 4.9], *t*_11_ = 5.80, *P* < 0.001, *dz* = 1.67).

#### Right leg (exercising leg)

No condition × time interaction was observed for right leg pain (*F*_2.90, 31.94_ = 0.965, *P* = 0.419, ${h}_{\mathrm{p}}^{2}$ = 0.081) or main effect of condition (*F*_1, 11_ = 1.053, *P* = 0.327, ${h}_{\mathrm{p}}^{2}$ = 0.087). There was a main effect of time (*F*_2.48, 27.31_ = 85.145, *P* < 0.001, ${h}_{\mathrm{p}}^{2}$ = 0.886). Pain intensity increased at every time point from 30 s to task failure (all *P* < 0.009) (Fig. 2b). There was also no difference in peak pain (CTRL = 9.0 [1.9], HYP = 9.0 [1.4] (Wilcoxon *P* = 0.371). There was no difference in the sum of the Situation Specific Pain Catastrophizing Scale post-TTF (*P* = 0.733).

### Maximal voluntary force

**Fig. 3:**
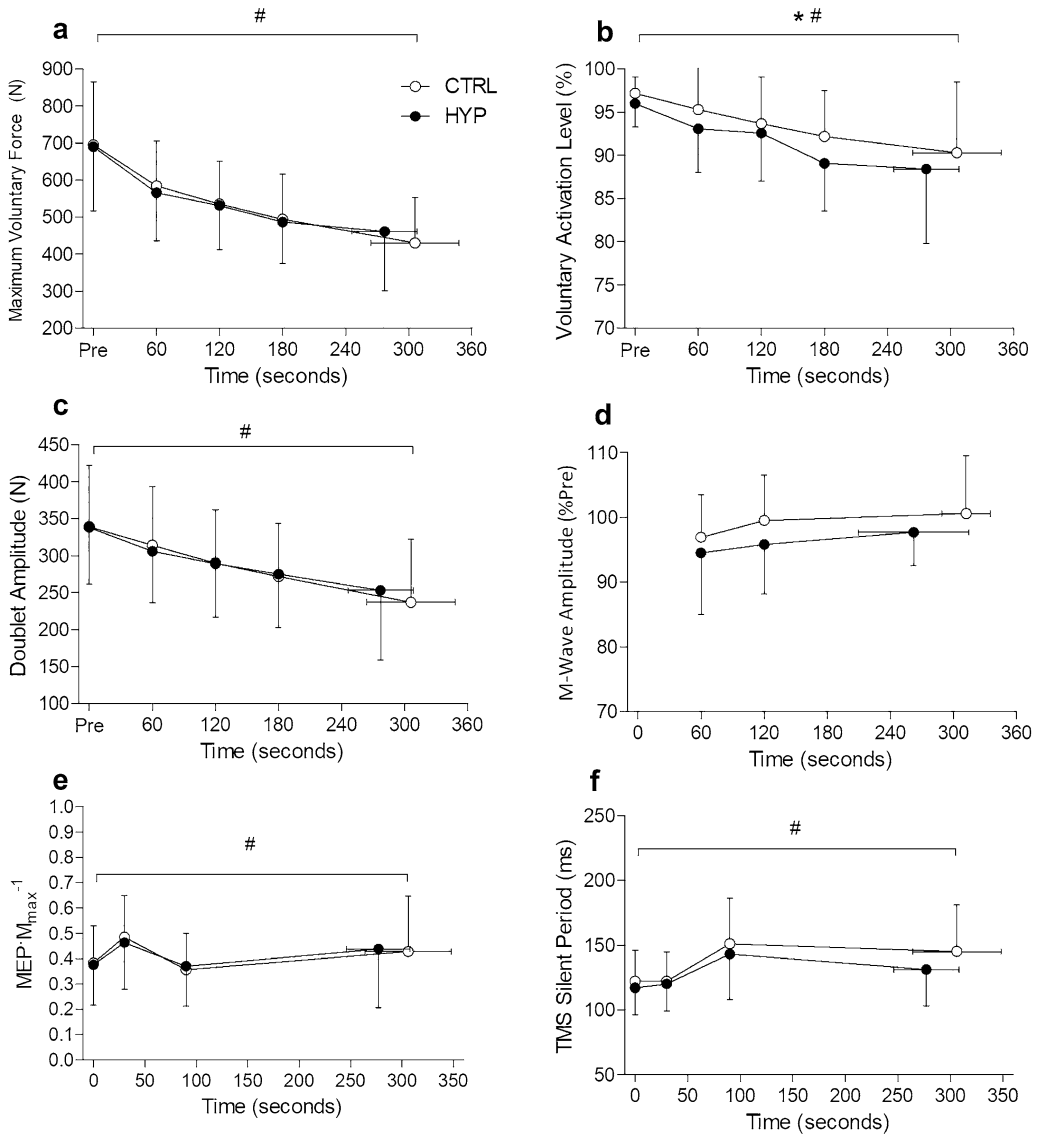
Neuromuscular variables during the isometric TTF. **a** Maximum voluntary force. **b** Voluntary Activation Level. **c** Doublet amplitude. **d** Change in *M*_max_. **e** Corticospinal excitability as MEP·*M*_max_^−1^. **f** Corticospinal inhibition as the TMS silent period. Data presented as mean ± SD. * denotes main effect of condition (*P* < 0.05). # Denotes main effect of time (*P* < 0.05)

For MVF there was no condition × time interaction (*F*_1.32 14.53_ = 1.90, *P* = 0.190, ${h}_{\mathrm{p}}^{2}$ = 0.147) or main effect of condition (*F*_1, 11_ = 0.002, *P* = 0.958, ${h}_{\mathrm{p}}^{2}$ = 0.001). However, there was a main effect of time (*F*_1.85, 20.40_ = 28.45, *P* < 0.001, ${h}_{\mathrm{p}}^{2}$ = 0.721). MVF decreased at each timepoint from baseline (692 ± 168 N) until task failure (446 ± 141 N) (all *P* < 0.05) (Fig. 3a).

### Voluntary activation

No condition × time interaction was observed for voluntary activation (*F*_1.57, 17.29_ = 0.312, *P* = 0.684, ${h}_{\mathrm{p}}^{2}$ = 0.162). However, there was a main effect of condition (*F*_1, 11_ = 6.029, *P* = 0.032, ${h}_{\mathrm{p}}^{2}$ = 0.354) and main effect of time (*F*_2.53, 27.87_ = 9.640, *P* < 0.001, ${h}_{\mathrm{p}}^{2}$ = 0.467). VA was lower in HYP compared to CTRL. Over time, VA decreased from baseline (96.6 ± 2.3%) to minute 3 (90.7 ± 5.4%) (*P* = 0.007) but did not decrease any further at task failure (89.4 ± 8.3%) (*P* = 0.334) (Fig. 3).

### Potentiated doublet

For potentiated doublet there was no condition × time interaction (*F*_2.13, 23.42_ = 2.638, *P* = 0.090, ${h}_{\mathrm{p}}^{2}$ = 0.193) or main effect of condition (*F*_1, 11_ = 0.159, *P* = 0.698, ${h}_{\mathrm{p}}^{2}$ = 0.014), but there was a main effect of time (*F*_1.38, 15.19_ = 28.923, *P* < 0.001, ${h}_{\mathrm{p}}^{2}$ = 0.724). Doublet amplitude decreased at every timepoint from baseline (338 ± 78 N) to task failure (245 ± 88 N) (all *P* < 0.05) (Fig. 3).

### *M*_max_

No condition × time interaction was observed for *M*_max_ (*F*_1.32, 14.48_ = 1.880, *P* = 0.193, ${h}_{\mathrm{p}}^{2}$ = 0.146). There was also no main effect of condition (*F*_1, 11_ = 3.250, *P* = 0.099, ${h}_{\mathrm{p}}^{2}$ = 0.228) or main effect of time (*F*_1.41, 15.51_ = 1.59, *P* = 0.233, ${h}_{\mathrm{p}}^{2}$ = 0.126).

### MEP·*M*_max_^−1^

**Fig. 4:**
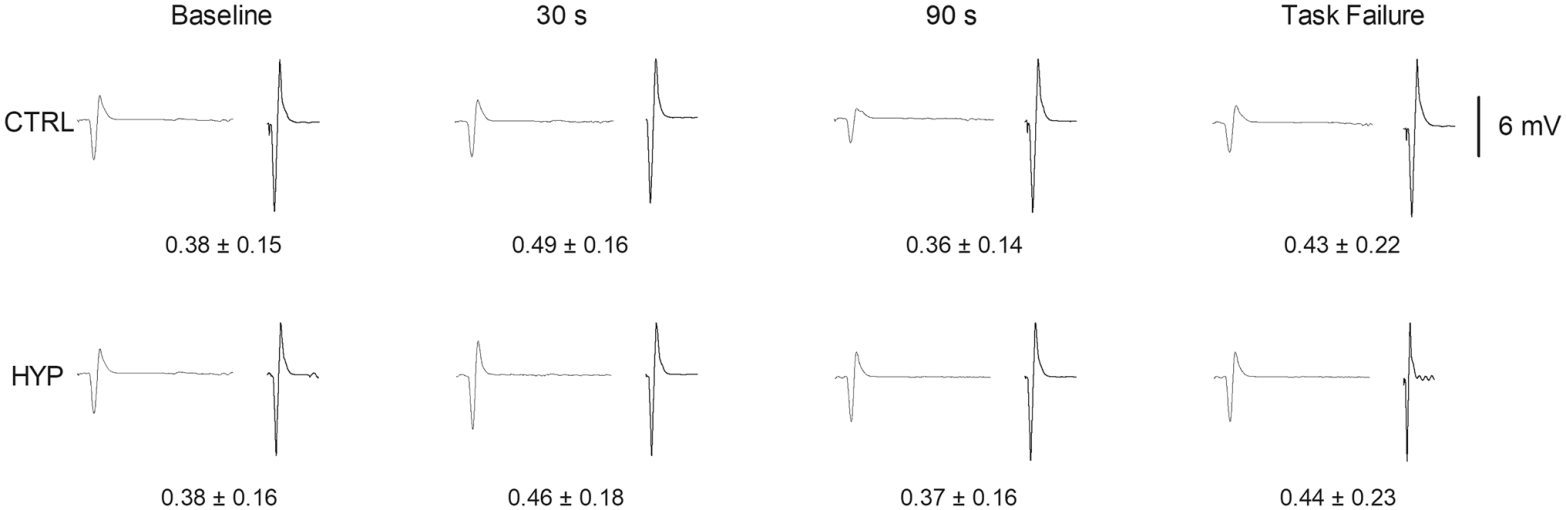
Representative traces of motor evoked potentials and M-Waves for each experimental condition at each time point. First trace is average of the MEPs and the second trace is the M-Wave

There was no condition × time interaction for MEP·M_max_^−1^ (*F*_3, 33_ = 1.147, *P* = 0.345, ${h}_{\mathrm{p}}^{2}$ = 0.094) or main effect of condition (*F*_1, 11_ = 0.006, *P* = 0.936, ${h}_{\mathrm{p}}^{2}$ = 0.001), but there was a main effect of time (*F*_3, 33_ = 7.866, *P* < 0.001, ${h}_{\mathrm{p}}^{2}$ = 0.417). MEP·*M*_max_^−1^ increased from baseline (0.38 ± 0.15) to 30 s (0.48 ± 0.17) (*P* = 0.001) and subsequently decreased from 30 to 90 s (0.36 ± 0.15) (*P* < 0.001) and then remained unchanged at task failure (0.43 ± 0.22; *P* = 0.178). Representative traces of MEPs and M-Waves can be seen in Fig. 4.

### TMS silent period

No condition × time interaction was seen for the TMS silent period (*F*_1.82, 20.01_ = 1.92, *P* = 0.176, ${h}_{\mathrm{p}}^{2}$ = 0.148) or main effect of condition (*F*_1, 11_ = 3.39, *P* = 0.093, ${h}_{\mathrm{p}}^{2}$ = 0.235). However, there was a main effect of time (*F*_1.46, 16.07_ = 7.92, *P* = 0.007, ${h}_{\mathrm{p}}^{2}$ = 0.419). The TMS silent period did not increase from baseline (120 ± 22 ms) to 30 s (121 ± 21 ms) (*P* = 0.836) but increased from 30 to 90 s (147 ± 34 ms) (*P* = 0.019) and did not change any further at task failure (138 ± 33 ms) (*P* = 0.105).

### Electromyography amplitude

#### MVCs

**Fig. 5:**
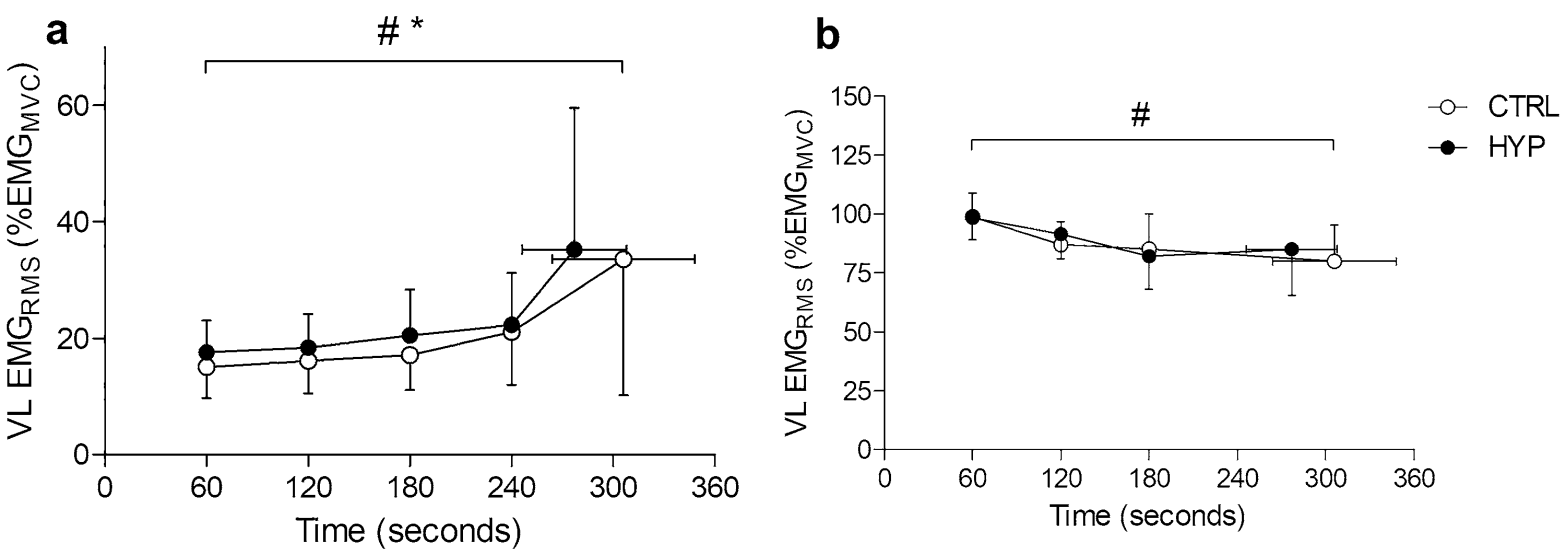
Root mean square electromyography amplitude during the isometric TTF. **a** Submaximal amplitude of the vastus lateralis muscle. **b** MVC EMG amplitude of the vastus lateralis * Denotes significant main effect of condition (*P* < 0.05). # Denotes significant main effect of time (*P* < 0.05)

No condition × time interaction was observed for MVC EMG_RMS_ amplitude (*F*_1.73, 19.01_ = 3.077, *P* = 0.076, ${h}_{\mathrm{p}}^{2}$ = 0.219). There was also no main effect of condition (*F*_1, 11_ = 0.920, *P* = 0.358, ${h}_{\mathrm{p}}^{2}$ = 0.077) but there was a main effect of time (*F*_1, 11_ = 10.592, *P* < 0.001, ${h}_{\mathrm{p}}^{2}$ = 0.491). MVC EMG_RMS_ amplitude decreased from minute 1 to minute 2 (*P* = 0.028,) but did not decrease further at minute 3 or at task failure (both *P* > 0.05) (Fig. 5).

#### Time to task failure

For EMG_RMS_ amplitude of the isometric TTF there was no condition × time interaction (*F*_2.19, 24.04_ = 0.847, *P* = 0.450, ${h}_{\mathrm{p}}^{2}$ = 0.071). However, there was a main effect of condition (*F*_1, 11_ = 11.983, *P* = 0.005, ${h}_{\mathrm{p}}^{2}$ = 0.521) whereby EMG_RMS_ was greater in HYP compared to CTRL. There was also a main effect of time (*F*_1.04, 11.48_ = 7.384, *P* = 0.019, ${h}_{\mathrm{p}}^{2}$ = 0.402). EMG_RMS_ increased from minute 1 (16.4 ± 5.4%) to minute 3 (18.8 ± 7.1%) (*P* = 0.047) and from minute 3 to minute 4 (21.7 ± 8.8%) (*P* = 0.036) but did not further increase at the point of task failure (34.4 ± 23.4%) (*P* = 0.083).

### Rating of perceived effort (RPE)

For RPE, no condition × time interaction effect was observed (*F*_3, 33_ = 0.791, *P* = 0.507, ${h}_{\mathrm{p}}^{2}$ = 0.067) and there was no main effect of condition (*F*_1, 11_ = 3.561, *P* = 0.086, ${h}_{\mathrm{p}}^{2}$ = 0.245). There was a main effect of time (*F*_1.83, 20.10_ = 148.689, *P* < 0.001, ${h}_{\mathrm{p}}^{2}$ = 0.930) whereby RPE increased at every timepoint from minute 1 (11 ± 2) to task failure (19 ± 1) (all *P* < 0.001).

## Discussion

The primary finding of this study is that acute muscle pain reduces endurance performance in the contralateral limb. This appears to be due an exacerbation of perceptual responses (i.e., left leg pain) along with a decrement to voluntary activation.

### Pain and TTF

The hypertonic saline injection caused a rapid increase in pain intensity of the left leg which peaked by 60 s at ~ 4.5/10 (somewhat strong pain), and then slowly decreased over time due to the gradual dissipation of the intramuscular saline. By the end of the TTF, the pain in the left leg was not significantly different from CTRL. As expected, in the exercising limb there was a gradual increase in pain intensity during the TTF, which reached near maximal levels. However, there was no difference in pain intensity of the right leg between HYP and CTRL, meaning that the pain in the exercising leg was unaffected by concurrent pain in the contralateral leg.

Only one other study, by Aboodarda et al. (2020), has investigated the impact of exclusively non-local muscle pain on endurance performance. They found a 21% reduction in TTF with concurrent rising pain compared to the control, almost two-fold greater than in the current study. This may be explained by the gradual increase of muscle pain in the contralateral limb due to the ischemic environment induced, whereas with a hypertonic saline injection, muscle pain rapidly increases then slowly decreases (i.e., Fig. 2). As a result, there was likely a greater summation of afferent feedback in the latter parts of the exercise in the Aboodarda study which was exerting a greater inhibitory effect on endurance performance.

### Neuromuscular fatigue

Despite a significant pain response in the left leg, the impact of pain on neuromuscular fatigue during the endurance task was limited. No difference was observed in maximum voluntary force between conditions. This is in contrast to work by others (Deschamps et al. 2014) who found a reduction in maximal hopping performance after a hypertonic saline injection into the contralateral vastus lateralis. However, it is difficult to draw direct comparisons between MVCs and maximal hopping efforts as there are differences in muscle activation and stability between tasks. On the other hand, when hypertonic saline was injected into iliotibial tract (targeting non-muscle nociceptors), a decrement in the force generating capacity of the contralateral knee extensors and ipsilateral hand grip muscles was observed (Oda et al. 2018). This was, however, observed with a greater pain intensity than achieved in this study (peak pain of 8.5) Whilst it cannot be ruled out that pain may reduce contralateral muscle strength, the findings of the present study do not suggest that a reduction in maximal force generating capacity was responsible for the shorter TTF. This is in contrast with localised muscle pain, which does appear to reduce maximal force generating capacity (Graven-Nielsen et al. 1997; Smith et al. 2020; Norbury et al. 2022).

There was a main effect of condition for voluntary activation whereby VA was lower in HYP compared to CTRL. Interestingly, this did not result in a decrease in the maximal force generating capacity of the knee extensors. It is not clear why this occurred, as peripheral fatigue also did not differ at this time point. The reduction in VA likely reflects a combination of neural inhibitory feedback from both limbs acting to constrain voluntary drive to the right knee extensor. In the right leg, inhibition would likely have been caused by the stimulation of fatigue sensitive and nociceptive group III/IV afferents from accumulation of metabolites, whereas in the left leg inhibition would have been driven purely by the pain related feedback from the nociceptors stimulated by the hypertonic saline. In combination, these caused a greater reduction in central motor output within HYP. The reason for this reduction in central motor drive is likely to prevent the attainment of an intolerable level of voluntary activity (Gandevia 2001; Hureau et al. 2018).

Post-exercise neuromuscular fatigue was not different between CTRL and HYP which is in contrast with several studies which have induced contralateral pain or fatigue and then performed a subsequent TTF (Amann et al. 2013; Johnson et al. 2015; Aboodarda et al. 2020). Neural inhibitory feedback which can ‘spill over’ from the non-local area is thought to cause an individual to reach their sensory tolerance limit at an accelerated rate (Hureau et al. 2018). A shortened TTF would result in less end-exercise neuromuscular fatigue, which is typically seen with an attenuated reduction in maximum voluntary force and peripheral fatigue. It is unclear in the current study because despite a reduction in TTF, there was no difference in end-exercise neuromuscular fatigue. Perhaps only a modest reduction in TTF observed in this study (~ 10%) was insufficient to cause a significant attenuation of end-exercise neuromuscular fatigue, whereas in prior studies reductions in TTF have been much greater (21–50% reduction) (Amann et al. 2013; Johnson et al. 2015; Aboodarda et al. 2020).

### TMS responses

The MEP·*M*_max_^−1^ ratio increased at 30 s then decreased at 90 s during the exercise task reflecting an increase in excitability early on in the exercise, before exercise-induced fatigue likely decreased the excitability of the corticospinal pathway (Finn et al. 2018). However, there was no observable difference in MEP·*M*_max_^−1^ between conditions. Therefore, the excitability of the cortico-spinal pathway was unaffected by non-local muscle pain. These findings are in agreement with Le Pera et al. (2001) who observed no effect of muscle pain on motor evoked potential amplitude of the contralateral hand. The TMS silent period, which is thought to reflect inhibition of the corticospinal pathway (Goodall et al. 2018; Škarabot et al. 2019), increased 90 s into the endurance task but was not further increased with the addition of non-local muscle pain. This is also in alignment with previous work (Aboodarda et al. 2020) where no increase of the TMS silent period with non-local pain and a shortening with non-local fatigue was observed. Therefore, when pain is non-local, it appears to have no influence on the cortico-spinal pathway during fatiguing exercise. However, this may not be the case for localised muscle pain where corticospinal adjustments may be responsible for changes in motor function (Schabrun and Hodges 2012).

### Electromyographic responses

An interesting finding in the present study was that the VL EMG amplitude during the TTF was greater in HYP than in CTRL. The EMG recorded during submaximal tasks are thought to provide a crude measure of the neural drive to the muscle (Farina et al. 2010). In this study, pain in the left VL could have subsequently required an increased neural drive to the right VL during the endurance task due to the centrally mediated inhibition caused by muscle pain (Farina et al. 2004; Liew et al. 2019; Martinez-Valdes et al. 2020) which could necessitate a greater need for central drive to ensure the maintenance of force. As a result, the earlier recruitment of more fatigable, higher threshold motor units could lead to the earlier development of fatigue and a shortened TTF.

### Sensory tolerance limit

The sensory tolerance limit (Hureau et al. 2018) postulates that sensory feedback from muscles not directly involved in the exercise and corollary discharge summates until an intolerable level is achieved which causes a decrease in voluntary activation and termination of exercise. Within this study, it appears that the additional sensory neural feedback in the non-exercised left leg combined with the rising exercise-induced fatigue and pain in the right leg to cause the individual to reach their sensory tolerance limit sooner and cause a premature task failure. Indeed, a reduced TTF for quadriceps exercise with the have occurred with additional sensory neural feedback from respiratory muscles (Wüthrich et al. 2013), contralateral quadriceps (Amann et al. 2013; Aboodarda et al. 2020) and upper body muscles (Johnson et al. 2015). The greater RPE in HYP within this study also supports this notion.

### Methodological considerations

The acute nature of the hypertonic saline injection resulted in a decreasing pain intensity within the left leg at the latter stages of the endurance task. It should be acknowledged that the neuromuscular and endurance performance reducing effects of non-local pain may have been more pronounced if a consistent or increasing level of pain was induced. Therefore, the effects in the present study are likely an underestimation of the role of non-local muscle pain on endurance performance.

Three females participated in this study which may introduce sex differences which could influence the results observed; particularly as we did not control for phases of the menstrual cycle or hormonal contraceptives. Firstly, in terms of the TTF intensity, it is likely that females performed the isometric TTF under different levels of ischemia compared to males due to (on average) a lower absolute strength (Males means absolute target = 145 N, Females = 118 N). This may influence the aetiology of pain and fatigue between the sexes and is acknowledged as a limitation within the present study. Furthermore, neurophysiological measures such as voluntary activation and short intracortical inhibition can change during different phases of the menstrual cycle, possibly due to differing levels of circulating oestrogen and progesterone. In regard to sex differences in pain perception, it appears males and females experience a similar intensity of pain from hypertonic saline injections (Loram et al. 2009; Yekkalam et al. 2019). However, we acknowledge that differing phases of the menstrual cycle and the use of oral contraceptives can also be important as the response to experimental pain has been shown to be influenced by the menstrual cycle phase (Sherman and LeResche 2006). Specifically, analgesia in the luteal phase (Vincent et al. 2018) may have caused differing levels of pain in response to exercise. However, in the exercising leg, pain was similar. Therefore, any influence of the menstrual cycle would have likely been minimal. Fortunately, only two experimental visits were performed which were no longer than 7 days apart for females which would minimise the chance of the experimental visit being completed at different phases of the menstrual cycle.

## Conclusion

In summary, muscle pain/nociceptive activity in a contralateral limb causes a significant reduction in endurance performance. This effect is centrally mediated and likely arises from a faster attainment of the sensory tolerance limit due to elevated levels of sensory neural feedback relayed from nociceptors in the painful left leg.

## Data Availability

Raw data are available upon request from the corresponding author.
